# Atosiban versus betamimetics in the treatment of preterm labour in Germany: an economic evaluation

**DOI:** 10.1186/1471-2393-9-23

**Published:** 2009-06-19

**Authors:** Jaro Wex, Mark Connolly, Werner Rath

**Affiliations:** 1 PharmArchitecture Limited, Quatro House, Lyon Way, Camberley, Surrey, UK; 2 Global Market Access Solutions, Ch. De Penguey 6B, St Prex, Switzerland; 3 Department of Gynaecology and Obstetrics, University Hospital RWTH, Aachen, Germany

## Abstract

**Background:**

The use of tocolytics is central in delaying birth; however, therapeutic options vary in effectiveness and adverse events profiles, which in turn could have consequences for medical resource use and cost of treatment.

Betamimetics are commonly used tocolytic agents, but their mechanism of action affects multiple organ systems leading to numerous adverse events. The availability of an oxytocin receptor antagonist, specific for prevention of preterm labour, offers a treatment option that merits further evaluation. We aimed to compare economic implications of tocolysis using atosiban and betamimetics, considering treatment efficacy and safety, as well as cost consequences of treatment of associated adverse events.

**Methods:**

A systematic literature review identified six randomised clinical trials, three of them double-blinded, comparing atosiban with betamimetics, in which tocolysis was initiated within 48 hours of admission. Cost of drug treatment was calculated based on trial protocols and German hospital drug purchase costs. G-DRG Grouper was used to obtain cost per case. The drug regimen was concordant with the German guidelines for the management of preterm labour, with two alternative modalities of fenoterol analysed: continuous or bolus administrations.

**Results:**

According to the results of the meta-analysis of the three double-blinded, placebo-controlled clinical trials, atosiban and betamimetics have similar efficacy (RR = 0.99, 95%CI:0.94–1.04, p = 0.772). Compared to betamimetics, use of atosiban was associated with a significantly lower frequency of adverse events for tachycardia, palpitation, vomiting, headache, hyperglycaemia, tremor, dyspnoea, chest pain, hypocalemia and foetal tachycardia.

In our economic analysis, cost savings from using atosiban versus continuous, or bolus, fenoterol was 423€ per patient from the payer's perspective. From the hospital's perspective, savings from using atosiban versus continuous fenoterol ranged from 259€ for 18 hours of tocolysis to 105€ for 48 hours; the respective values for bolus fenoterol were 244€ and 55€. In the probabilistic sensitivity analysis atosiban was cost saving versus both continuous and bolus fenoterol in 87%–100% of scenarios.

**Conclusion:**

In a German setting, atosiban is cost saving versus betamimetics in the treatment of preterm labour from the payer, hospital and combined perspectives. Cost savings stem from the superior safety profile of atosiban.

## Background

Preterm birth, defined as birth prior to completion of 37 weeks of gestation [1], occurs in 5–12% of all pregnancies in industrialised countries [2,3]. In addition to the enormous negative psychosocial and emotional burden on the family, preterm birth is associated with significant cost implications for society. In the USA alone annual cost of $26.2 billion or $51,600 per infant, has been attributed to preterm birth [4].

Despite intensive worldwide efforts to reduce the preterm birth rate, over the last 10 years it has risen in Germany to 9% of all pregnancies [5]. This reflects a 20% increase over the same period. Similarly, in the USA there has been a noticeable 38% increase in preterm deliveries since 1981 to reach 12.7% of all pregnancies [5].

Preterm birth accounts for 75% of all causes of perinatal mortality and the total risk for childhood disabilities is 10–15% for births before the 37^th ^week of gestation. Moreover, sixty five percent of children born before the 26^th ^week of gestation will not survive, and only 13% will survive for 30 months without a handicap [6,7].

The onset of preterm labour is believed to be a syndrome initiated by multiple mechanisms, including intrauterine infection or inflammation, uteroplacental ischemia or haemorrhage, uterine over-distension, stress, or other immunologically mediated processes [4]. Due to the multifactorial origin, treatment options generally target the inhibition of uterine contractility, rather than the underlying mechanisms of preterm labour.

Tocolytics, used in Europe for the last 30 years, are the backbone of treatment for the prevention of preterm labour. Tocolytic drugs are indicated to prolong pregnancy in women with acute risk of preterm birth; in some cases, they are also administered for prophylaxis. The main rationale for use of these drugs is to delay delivery for at least 48 hours in order to allow time for the treatment effect of corticosteroids, or transfer of the pregnant mother to a specialized high-risk obstetrical unit [8]. Both actions have been shown to markedly reduce neonatal morbidity and/or mortality.

As most randomized trials (RCTs) of tocolytic agents lack power to provide definitive answers, and use delay in delivery as a surrogate endpoint for implied improvements in neonatal outcome related to the administration of steroids, systematic reviews and meta-analyses have been conducted to more adequately study drug efficacy and safety. Several Cochrane reviews have been published that compared different tocolytic agents to each other or to placebo for women in preterm labour [9-14]. Of these medications, cyclo-oxygenase (COX) inhibitors were considered to have an inadequate knowledge base to be recommended for use [9]. Magnesium sulphate was demonstrated to be an ineffective tocolytic agent, with an increased risk of infant mortality [10]. Prophylactic oral betamimetics for preventing preterm birth in women at high risk of preterm labour with a singleton pregnancy were inadequately studied to provide any definitive answers [11], but betamimetics in general have been shown to delay delivery [12]. Even so, multiple adverse events must be considered prior to its explicit recommendation. Nifedipine was shown to be preferable to other tocolytic agents regarding maternal and neonatal outcomes, but no placebo-controlled trials were available to reviewers [13]. Finally, atosiban was considered to be equivalent to betamimetics in preventing preterm labour [14].

The choice of tocolytic agent depends mainly on the availability following regulatory approval in the country, the drug efficacy and effectiveness, the foetal and maternal safety profiles, and the costs of treatment. In Germany, only atosiban, fenoterol, and magnesium sulphate (in limited situations) are approved for treatment of preterm labour [15].

Betamimetics are commonly used in tocolysis, but since their mechanism of action is organ non-specific, they notoriously lead to numerous adverse events in multiple organ systems, which are largely of cardiovascular origin. With similar efficacy, the benefits of atosiban result from the significantly better safety profile with a significantly lower rate of foetal and maternal side effects and a significantly lower rate of treatment discontinuation [16].

Many centres use nifedipine, a calcium channel blocker, as a relatively inexpensive, orally administered tocolytic. In the Cochrane review comparing calcium channel blockers to other tocolytic agents, mainly betamimetics, the former was shown to be an effective treatment option [13]. Even so, due to the lack of regulatory approval in Germany, and most other countries, nifedipine is only being used off-label for the treatment of preterm labour.

A recent study by Hayes et al. [17] reported the probability of adverse events (AEs) for various tocolytic treatments and the costs attributed to AEs in the United States. Because treatment practices and reimbursement mechanisms in preterm labour differ between Europe and the US, we undertook an economic evaluation of tocolytic therapy for the treatment of preterm labour comparing atosiban with betamimetics using a study design similar to Hayes et al., but incorporating the German cost data in a system of payer-provider split.

## Methods

We systematically searched MEDLINE, EMBASE, the Centre for Reviews and Dissemination (CRD) and the Cochrane Central Register of Controlled Trials (CENTRAL) databases to identify randomized clinical trials comparing atosiban versus betamimetics in women experiencing preterm labour. No search restrictions were applied and all abstracts identified using the keywords "atosiban", "Tractocile", "antocin", and/or "RWJ 22164" were considered. For the meta-analysis of efficacy and rates of adverse events, we included trials that provided outcomes during 48 hours of hospitalisation. Three types of outcomes data were extracted from the selected studies: efficacy in delaying preterm birth by at least 48 hours, frequency of maternal and foetal adverse events, and resource utilisation for the economic analysis. The inclusion criteria in the identified three double-blinded studies were: regular preterm uterine contractions, cervical dilatation of 0–3 cm (for nulliparae) or 1–3 cm (for multiparae), cervical effacement of ≥50%, ≥18 years of age or of legal consenting age, gestational age between 23–33 weeks (confirmed by ultrasound before 20 weeks and/or reliable menstrual dates). Exclusion criteria were high-order multiple pregnancy greater than twins, ruptured membranes, major vaginal bleeding, severe pre-eclampsia or hypertension, fever (body temperature >37.58C), urinary tract infection, fetal/placental abnormalities, serious maternal disease, any contraindication to the use of beta-agonists, alcohol or drug abuse, history of hypersensitivity to any component of the study drugs, previous exposure to any tocolytic therapy within six hours (or within 12 hours for indomethacin) of study entry, and participation in a clinical trial of an experimental drug within the previous month. The inclusion and exclusion criteria in the remaining three studies differed from the above in minor details. The study quality characteristics of all identified trials are presented in a supplementary table [see Additional file 1 – STable 1].

In Germany, fenoterol is the only betamimetic agent approved for use in preventing preterm labour [15]. To the best of our knowledge, and confirmed by our systematic search, no clinical trials comparing atosiban directly with fenoterol exist. Even so, previous analyses of adverse events reported in trials of atosiban versus three different betamimetics demonstrated comparable safety profiles of the latter [18]. Therefore, we did not limit our review of the evidence to fenoterol, but included all betamimetic agents, as their efficacy and safety profiles were expected to be similar. Using the Mantel-Haenszel method (fixed-effect model), we performed evidence synthesis using a dedicated statistical package [19].

A cost-minimisation analysis, rather than a cost-effectiveness analysis, was conducted due to the similar efficacy of the analysed tocolytic treatments. A review of resource utilisation and expert opinion revealed that costs related to drug administration were not different and could not be captured in the German payment system. Diagnostic costs attributable in cases when the patient was ineligible for betamimetics (e.g., high cardiovascular risk, diabetes, thyrotoxicosis) were not considered, as in such cases alternative tocolytic treatment is typically preferred in clinical practice instead of diagnostic testing. Finally, regardless of the tocolytic agent, we assumed that the costs of monitoring of tocolysis, as well as all other costs related to management of preterm labour and preterm birth would be the same in all women experiencing preterm labour. Drug costs were evaluated at 18 and 48 hours from the time of hospital admission. For increased accuracy, extended hospitalisation for treatment of emergent adverse events occurring within the 48 hours period was also factored into the analysis.

Costing methodology was guided by the German Diagnosis Related Group (G-DRG) method of financing of inpatient treatments used in the German health care system. DRGs are essentially homogeneous average costs groups, often adjusted for case severity. A certified G-DRG Grouper [20] was used to calculate cost of treatment of preterm labour and any associated adverse events of tocolysis.

From the payer's (social health insurer's) perspective, all costs associated with treatment of preterm labour were encompassed by the flat DRG rate per diagnosed patient. From this perspective, only extended length of stay and occurrence of chest pain or dyspnoea had cost consequences resulting from DRG recoding.

From the hospital's perspective, every extension of length of stay had cost implications for the hospital, even if no DRG recoding was possible and no additional payments were due from the payer. DRG tariff was used to obtain the average cost of a hospitalisation day, based on the premise that DRG reflects all diagnosis related costs, including overheads. Since costs of tocolytic drugs were included in the DRGs, the use of a more expensive drug generated additional costs for the hospital, whereas the analysis from the payer's perspective did not involve drug costs. Costs of atosiban (Tractocile) and fenoterol (Partusisten) were obtained from the hospital purchase price list [Ferring, data on file] and the Rote Liste [21], respectively. VAT of 19% was added to the hospital purchase price. We also calculated the costs from the combined perspective, theoretical in a system with a payer-provider split, but justified from the economic point of view. In this approach, cost of tocolytic drugs, daily cost of extended hospitalisations, and cost of treatment of adverse events were added. Details of all cost variables used in the analysis are shown in Table 1.

**Table 1:** Cost variables used as input in the economic model.

**Variable**	**Value**	**95% uncertainty range**	**Reference**	**Comment**
Atosiban (Tractocile) 6.5 mg	35.47€	31.93–39.02€	Ferring, data on file	Costs of diagnostics, monitoring and infusion fluids considered equal for all treatments; drug wastage accounted for; 19% VAT applied.
Atosiban (Tractocile) 37.5 mg	115.29€	103.76–126.82€	Ferring, data on file	Costs of diagnostics, monitoring and infusion fluids considered equal for all treatments; drug wastage accounted for; 19% VAT applied.
Fenoterol (Partusisten) 0.5 mg	3.75€	3.37–4.12€	Rote Liste [14]	Costs of diagnostics, monitoring and infusion fluids considered equal for all treatments; drug wastage accounted for; 19% VAT applied.
Preterm labour, one hosp. day	321.90€	Fixed value	G-DRG 2007/2008 [13]	G-DRG O64B
Preterm labour, more than one hosp. day	1,742.90€	Fixed value	G-DRG 2007/2008 [13]	G-DRG O64A
Chest pain	1,175.50€	Fixed value	G-DRG 2007/2008 [13]	G-DRG FZ4Z, min. 2 hosp. days were assumed for cases with chest pain.
Dyspnoea	2,470.80€	Fixed value	G-DRG 2007/2008 [13]	G-DRG E64D, min 2 hosp. days were assumed for cases with dyspnoea.

The dosing regimen used for the calculation of costs of drugs was based on the protocols used in the included clinical trials [22-24]. Atosiban was administered i.v. in a single bolus dose (6.75 mg in 0.9 mL normal saline), with subsequent infusion of 300 mcg/min atosiban in 5% dextrose for the first 3 hours, followed by 100 mcg/min atosiban in 5% dextrose for up to 48 hours. The regimen was concordant with the recommendation of the German guidelines for the management of preterm labour [15]. The dosage of fenoterol was adopted from the German guidelines, with two alternative modalities analysed: continuous or bolus administrations. In the continuous regimen, fenoterol was started at 2 mcg/min, increasing the dose by 0.8 mcg/min every 20 min; maximum 4 mcg/min. Bolus tocolysis involved an initial dose of 10 mcg every 3 min with doubling the interval after contractions stopped (assumed after 1 hour), and further doubling of the interval after 12 and 24 hours. The option of initial increase of the bolus dosage when tocolysis was not effective was, conservatively, not considered. Moreover, drug wastage was accounted for in all scenarios.

The cost-minimisation analyses were conducted using an economic model developed by the authors in a Microsoft Excel spreadsheet; the model accounted for the payer-provider split characteristic of the German system. In the model, a cohort of 1,000 patients was followed up for up to 48 hours of hospitalisation for preterm labour. Tocolytic treatments were assigned based on the all-patients treated population from the combined clinical trials. Discontinuation of drug administration due to adverse events, progression of labour, preterm delivery and other causes was incorporated in the model. Weibull survival curves were fitted to the data on discontinuation at 48 hours, preterm delivery at 48 hours, and at 7 days. Drug switching from atosiban to fenoterol or from fenoterol to another betamimetic was also modelled, based on outcomes from the clinical trials. Drug switching was assumed to occur with equal probability during the 48 hours hospitalisation period. Hospitalisation length was defined based on expert opinion using beta distribution with the mean of 2.2 days, minimum of 1 day and maximum of 10 days. Occurrence of adverse events was associated with risk of extension of the hospitalisation length. It was assumed that on average only 50% (varied from 20 to 90% in the sensitivity analysis) of the patients experiencing any of the adverse events would require hospitalisation extended by one or more days. Occurrence of multiple adverse events was assumed to have the same consequences as occurrence of any single event. Effectively, we conservatively assumed no consequences of occurrence of adverse events in 50% of the patients. Similarly, diagnosis of chest pain or dyspnoea could lead to DRG recoding in 50% of the patients, with the same variation in the sensitivity analysis applied. In the sensitivity analysis, 95% uncertainty intervals were also estimated for efficacy, risk of adverse events and unit cost variables.

## Results

Based on the three double-blinded, placebo-controlled trials [22-24], efficacy of atosiban in delaying preterm birth by at least 48 hours was found to be identical to that of betamimetics (88.1% versus 88.7%, respectively; RR = 0.99, 95%CI:0.94–1.04, p = 0.772). Addition of a single-blinded [25] and two open label [26,27] trials resulted in slightly higher success rates in both treatment groups (90.2% versus 90.0%, respectively), but also yielded no significant difference (RR = 1.00, 95%CI:0.97–1.03, p = 0.965).

Meta-analysis of the three double-blinded, placebo-controlled clinical trials revealed, that the use of atosiban was associated with a significantly lower frequency of adverse events compared to betamimetics for six out of 16 adverse events reported. Inclusion of all six studies yielded a similar safety profile advantage of atosiban with five out of 16 adverse events occurring with significantly lower frequency. Disaggregated meta-analysis results for each adverse event for the three double-blinded trials, as well as all six included trials are detailed in Tables 2 and 3.

**Table 2:** Reported adverse events in the double-blinded, placebo-controlled trials only.

Adverse event	Frequency (%) Atosiban	Frequency (%) Betamimetics	Relative risk and Confidence Intervals	p-value
Tachycardia	5.50%	75.50%	RR = 0.07, 95% CI = 0.05, 0.11	<0.001
Palpitation	2.20%	15.60%	RR = 0.15, 95% CI = 0.07, 0.3	<0.001
Vomiting	6.90%	21.80%	RR = 0.31, 95% CI = 0.21, 0.48	<0.001
Headache	9.70%	18.60%	RR = 0.52, 95% CI = 0.35, 0.76	<0.001
Hyperglycaemia	6.40%	12.40%	RR = 0.53, 95% CI = 0.38, 0.85	0.008
Tremor	1.40%	15.90%	RR = 0.09, 95% CI = 0.04, 0.22	<0.001
Nausea	11.90%	15.90%	RR = 0.75, 95% CI = 0.52, 1.08	0.121
Dyspnoea	0.30%	7.30%	RR = 0.07, 95% CI = 0.02, 0.3	<0.001
Chest pain	1.10%	4.80%	RR = 0.22, 95% CI = 0.08, 0.65	0.006
Hypokalemia	0.80%	6.50%	RR = 0.15, 95% CI = 0.05, 0.45	<0.001
Hypotension	3.30%	5.70%	RR = 0.59, 95% CI = 0.29, 1.16	0.127
Anxiety	1.10%	2.40%	RR = 0.46, 95% CI = 0.15, 1.48	0.195
Syncope	0.60%	0.50%	RR = 1.02, 95% CI = 0.17, 5.98	0.984
Pulmonary oedema	0.30%	0.50%	RR = 0.6, 95% CI = 0.08, 4.45	0.614
Myocardial ischemia	0.00%	0.30%	RR = 0.34, 95% CI = 0.01, 8.3	0.509
Foetal tachycardia	3.30%	27.70%	RR = 0.13, 95% CI = 0.07, 0.26	<0.001

**Table 3:** Reported adverse events in all included trials

Adverse event	Frequency (%) Atosiban	Frequency (%) Betamimetics	Relative risk and Confidence Intervals	p-value
Tachycardia	3.90%	56.30%	RR = 0.07, 95% CI = 0.05, 0.1	<0.001
Palpitation	2.40%	20.40%	RR = 0.12, 95% CI = 0.06, 0.22	<0.001
Vomiting	5.90%	19.20%	RR = 0.27, 95% CI = 0.18, 0.4	<0.001
Headache	8.40%	14.10%	RR = 0.59, 95% CI = 0.43, 0.8	<0.001
Hyperglycaemia	7.80%	13.30%	RR = 0.57, 95% CI = 0.4, 0.89	0.001
Tremor	0.80%	12.20%	RR = 0.08, 95% CI = 0.04, 0.19	<0.001
Nausea	8.70%	10.50%	RR = 0.79, 95% CI = 0.57, 1.08	0.135
Dyspnoea	0.60%	8.50%	RR = 0.09, 95% CI = 0.04, 0.23	<0.001
Chest pain	1.70%	8.00%	RR = 0.18, 95% CI = 0.09, 0.38	<0.001
Hypokalemia	0.70%	7.10%	RR = 0.13, 95% CI = 0.05, 0.36	<0.001
Hypotension	2.80%	5.00%	RR = 0.6, 95% CI = 0.29, 1.11	0.1
Anxiety	1.50%	3.80%	RR = 0.43, 95% CI = 0.2, 0.91	0.027
Syncope	0.60%	0.50%	RR = 1.02, 95% CI = 0.17, 5.98	0.984
Pulmonary oedema	0.30%	0.50%	RR = 0.6, 95% CI = 0.08, 4.45	0.614
Myocardial ischemia	0.00%	0.30%	RR = 0.34, 95% CI = 0.01, 8.3	0.509
Foetal tachycardia	2.90%	21.10%	RR = 0.14, 95% CI = 0.08, 0.23	<0.001

**Figure 1:**
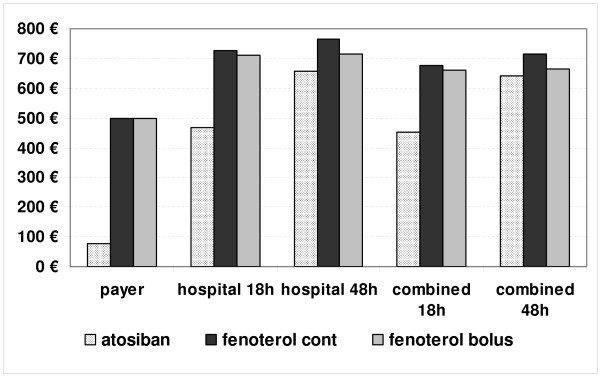
**Cost results for the three perspectives for different time horizons based on evidence from the three double-blinded clinical trials**.

Cost results for the three treatment options based on the double-blinded clinical trials are presented in Figure 1. Cost-minimization analysis revealed, that from the payer's perspective, cost savings from using atosiban versus fenoterol were 423€ per patient. From the hospital's perspective, savings from using atosiban versus continuous fenoterol ranged from 259€ for 18 hours of tocolysis to 105€ for 48 hours, and the respective values for bolus fenoterol were 244€ and 55€.

From the combined perspective, using atosiban versus continuous fenoterol saved from 226€ for 18 hours of tocolysis to 71€ for 48 hours, and versus bolus fenoterol the results were 211€ and 21€, respectively, jointly for the payer and hospital.

When all six RCTs were considered, cost savings from the payer's perspective using atosiban versus fenoterol were 371€ per patient. From the hospital's perspective, savings from using atosiban versus continuous fenoterol ranged from 217€ for 18 hours of tocolysis to 62€ for 48 hours, and the respective values for bolus fenoterol were 202€ and 13€. From the combined perspective, using atosiban versus continuous fenoterol saved from 210€ for 18 hours of tocolysis to 56€ for 48 hours, and versus bolus fenoterol the results were 195€ and 6€, respectively.

In the probabilistic sensitivity analyses, using data from the double-blinded trials, cost-savings for the payer varied in the 1,000 scenarios from 365€ (2.5 percentile) to 473€ (97.5 percentile) per patient. When all six clinical trials were used in the evidence synthesis, the respective values were 323€ and 431€. For both the hospital and combined perspectives, atosiban was cost saving versus both continuous and bolus fenoterol after 18 hours of tocolysis in 100% of scenarios. At 48 hours, atosiban was cost saving versus continuous fenoterol in 94.4% (89.6% for all six studies) of cases and versus bolus fenoterol in 87.2% (83.5% for all six studies) of cases. The respective values from the combined perspective were 93.0% (91.1% for all six studies) and 88.2% (85.7% for all six studies).

## Discussion

To the best of our knowledge, our study is the first economic evaluation of atosiban versus betamimetics based on evidence of efficacy and safety from a systematic literature review, and assessing the differential costs of tocolysis from the payer's and provider's perspectives. Heinen-Kammerer et al. [28] previously evaluated atosiban versus fenoterol in the German setting. This study merits close assessment as its results were later used for discussion in the context of implications for clinical practice [29]. Continuous fenoterol alone, continuous fenoterol with magnesium sulphate and bolus fenoterol were used as comparators in the decision analytic model. Success rates in delaying preterm birth by at least 48 hours were chosen as the measure of effectiveness, but only point estimates of success rates were considered in the model (0.770 vs. 0.670 vs. 0.626, respectively), with statistically significant differences implicitly assumed. The evidence base of the analysis did not appear comprehensive, as no study of atosiban versus betamimetics was considered. For atosiban and bolus fenoterol only one randomized clinical trial for each option was used as the source of efficacy data, while for continuous fenoterol tocolysis, a cohort study from the 1970s was chosen. Moreover, the study of atosiban had potentially biased outcomes due to imbalance in randomization with respect to gestational age. The authors in their model incorporated only one adverse event, pulmonary oedema. Our meta-analysis revealed that with regard to pulmonary oedema the compared options were not significantly different. In contrast, for ten other emergent adverse events the outcomes did differ.

Most importantly, Heinen-Kammerer et al. [28] added to the calculation the costs of preterm birth and its consequences for up to five years, which favoured fenoterol over atosiban with no justification in statistical significance of differential efficacy. The authors found that continuous fenoterol was 509€ more expensive than atosiban. Bolus fenoterol, however, was 1,191€ less expensive in comparison with atosiban; it was also 1,700€ less expensive than continuous fenoterol.

Our analysis of drug costs showed that the continuous regimen costs only 49€ more than bolus administration of fenoterol. Due to the markedly higher success rates for bolus fenoterol than that for continuous fenoterol (77.5% vs. 62.6%, respectively), assumed by Heinen-Kammerer at al. [28], most of the cost differential resulted from maternal, neonatal and paediatric treatments, as frequency of adverse events were assumed identical for the two fenoterol regimens. In contrast, with the scope of analysis limited to the costs of drugs and treatment of adverse events, adopted in our study, Heinen-Kammerer et al. [28] would have demonstrated cost savings with atosiban comparable, or greater, than our results not only for continuous, but also for bolus fenoterol. In the sensitivity analysis, the authors found that their results were highly dependent on the values of success rates used as input in the decision model. Based on this sensitivity, they concluded that no treatment option could be indicated as the preferred one. Our model based on input from the statistical analysis of the best available evidence, demonstrated that superiority of atosiban was robust in a sensitivity analysis and clear recommendation for clinical practice in Germany can be made for the comparison of these two tocolytics.

Both the analysis of Heinen-Kammerer et al. [28] and our evaluation consider bolus tocolysis with fenoterol as a valid treatment option. This regimen is used in 20–30% of German clinics [29]. No clinical trials of bolus fenoterol versus atosiban have been identified, and results from our analysis should be viewed considering the assumptions made with regard to the relative efficacy and safety of continuous and bolus fenoterol. Differences in efficacy of different tocolytic treatment options was, in turn, demonstrated between early and standard administration of atosiban, when pre-defined diagnostic eligibility criteria were met [30]. Inclusion of the immediate treatment option, which leads to higher success rates (88.9% vs. 76.1%, respectively), in our decision analytic model, would warrant considering maternal, neonatal and paediatric costs, as in the Heinen-Kammerer et al. [28] model. Given comparable safety profiles of the two atosiban arms, we can predict, that early tocolysis with atosiban would produce even greater cost savings when compared to betamimetics.

Atosiban was compared to another betamimetic, ritodrine, in the Spanish setting [31]. The decision-analytic model was based on the results of a clinical trial of atosiban versus ritodrine [24]. The average cost per patient treated with atosiban was 364€ greater than with ritodrine. Atosiban was judged dominated (more expensive and less effective), but the assumption of higher efficacy of ritodrine was not grounded in statistical significance. For the same reason, inclusion of pulmonary oedema among the adverse events was not warranted, while no other adverse events were considered in the analysis.

Another economic evaluation of atosiban versus a betamimetic was conducted in the Czech Republic [32]. This study was based on the same double-blinded clinical trials as ours [22-24], and an assumption of the equivalence of safety profiles of betamimetics used in the studies, of which none of them are approved for tocolysis in the Czech Republic, and fenoterol was made. The authors found, that in the Czech setting, the cost of treatment with atosiban or fenoterol were comparable at 18 hours, and atosiban became more expensive for the longer time horizon up to 48 hours. Since the reimbursement system could be critical in economic analysis (DRG, per diem, per procedure), applicability of results in different settings can be limited.

Established for Germany cost savings of 423€ per patient treated could have significant budget implications for the payer, if all eligible women are initiated on atosiban rather than on fenoterol. With the annual number of preterm births in Germany greater than 50,000, and based on the assumption that only half of women who deliver preterm are treated with tocolytics, the annual savings could be in excess of 20 million euros for the payer or 2–12 million for the hospitals. The savings would apply even given that 50–70% of women with symptoms of preterm labour deliver at term.

It is likely that our simplifying assumptions led to the underestimation of the cost savings potentially achieved with atosiban. We considered only direct medical costs associated with adverse events. While productivity loss resulting from extension of hospitalisation length may be of little importance in the context of consequences of preterm labour, direct non-medical costs could be non-trivial. In addition, the disutility resulting from adverse events was not included in the model.

Exclusion of diagnostic costs was also conservative. In an economic evaluation of four tocolytics drugs in the US, costs of cardiac evaluation, electrolyte monitoring and strict fluid status by means of Foley catheter were included for the betamimetic arm (terbutaline), because of the risk of maternal adverse events [17]. Higher incidence of foetal tachycardia could also lead to increased intensity of cardiotocographic monitoring with additional cost implications. Based on the three high quality studies, there was a trend for higher risk of caesarean section deliveries in the betamimetics group than in the atosiban group, but the difference was not significant at the 95% level (p = 0.059).

In the clinical trials used as the evidence base for the decision model, patients discontinuing treatment with a betamimetic received an alternative betamimetic agent. In our model, we assumed no cost consequences of such change, as no other betamimetic is available for tocolysis in Germany. If such patients were to be administered atosiban instead, the cost advantage per patient initiated on this drug would have been greater.

We based our analysis of comparable efficacy and safety profiles of fenoterol and other betamimetics. No clinical trial comparing atosiban to fenoterol was identified, but previously fenoterol showed similar safety profile to ritodrine for maternal tachycardia and hypoglycaemia [33]. For individual adverse events, ritodrine was, in turn, inferior to terbutaline for hyperglycemia or an abnormal glucose tolerance test and headache, and inferior to hexoprenaline for palpitations, hypotension, nausea and vomiting and foetal tachycardia. If the safety profile of fenoterol is similar to that of ritodrine, and clinical trials suggest inferiority of ritodrine in comparison to other betamimetics, our approach was conservative, with a possibility of underestimating the risk of adverse events on fenoterol. Other adverse events reported in trials of betamimetics versus placebo, such as cardiac arrhythmias (not significant) and nasal stuffiness (significant) [34], which could potentially occur with fenoterol, were not considered in the analysis.

Our cost analysis was subordinated to the reimbursement mechanisms based on the G-DRG tariff, rather than on detailed analysis of resource utilization and micro-costing. It was assumed that additional procedures associated with the treatment of adverse events do not affect the payer, as one of the reasons for using DRG is to enforce efficiency by shifting the financial risk to the provider, who is responsible for resource utilisation. In the longer term, however, inefficiencies at the provider level, would affect payer's budget, and detailed analysis of clinical resource utilisation should be informative for all stakeholders of the health system.

Results of a comparison of four tocolytics agents in the United States: nifedipine, magnesium sulphate, terbutaline and indomethacin had demonstrated that the latter two drugs had superior safety profile and were the cheaper treatment options than the comparators. Interestingly, these two superior drugs had been shown not to be popular treatment options in the United States [35], with 69% of women receiving magnesium sulphate. Similarly, in the German stetting, we have demonstrated that atosiban is not only clinically, but also economically superior to fenoterol, with the latter still being most widely used as the first line treatment option [28].

## Conclusion

In conclusion, in a German setting and according to German practice guidelines and treatment costs, atosiban was shown to be the preferred treatment option owing to cost savings resulting from its superior safety profile. As treatment safety is one of the clinical priorities, and in light of the fact that the resources are limited, we envisage that best available evidence combined with costing studies will be increasingly considered in making recommendations and decisions in preterm labour.

## Abbreviations

AE: Adverse event; CENTRAL: Cochrane Central Register of Controlled Trials; CRD: Centre for Reviews and Dissemination; G-DRG: German Diagnosis Related Group; RCT: Randomised Controlled Trial; VAT: Value Added Tax.

## Competing interests

JW is a director of an independent consultancy, which received an unrestricted research grant from Ferring Pharmaceuticals to study cost-effectiveness of tocolytic treatment options. MC is currently an independent consultant, and formerly, when the study was initiated, was an employee of Ferring Pharmaceuticals. WR was not remunerated for his contribution and declares no competing interests.

## Authors' contributions

JW conceived of the analytical approach, contributed to the literature review, designed the economic model and drafted the manuscript. MC contributed to the acquisition and analysis of data and to the review of the economic model. WR contributed to the analysis and interpretation of data and provided clinical information for costing study. All authors revised the manuscript and have given their final approval for submission.

## Pre-publication history

The pre-publication history for this paper can be accessed here:



## Supplementary Material

Additional file 1 **STable 1 – Characteristics of studies included in the analyses**. The data provides additional details on quality characteristics of studies included in the analyses. Click here for file
