# Supplementary material for: Atosiban versus betamimetics in the treatment of preterm labour in Germany: an economic evaluation
Source: BMC Pregnancy Childbirth. 2009 Jun 19;9:23. doi: 10.1186/1471-2393-9-23 (PMC2708127; doi:10.1186/1471-2393-9-23)
Supplement: Additional file 1 — STable 1 – Characteristics of studies included in the analyses. The data provides additional details on quality characteristics of studies included in the analyses. [file 1471-2393-9-23-S1.doc]

## STable 1 – Characteristics of studies included in the analyses.

|  | | **European 2001**  **[22]** | **French/ Australian 2001**  **[23]** | **Goodwin 1996**  **[26]** | **Husslein 2007**  **[27]** | **Moutquin 2000**  **[24]** | **Shim 2006**  **[25]** |
| --- | --- | --- | --- | --- | --- | --- | --- |
| (1) **Randomization sequence generation and allocation concealment**: | (a) Method of randomization sequence generation: | Computer-generated randomization lists stratified by clinical centre and GA | Computer-generated randomization lists stratified by GA | Computer-generated randomization lists stratified by clinical centre | Not reported | Computer-generated randomization lists stratified by GA | Computer-generated randomization lists stratified by GA |
|  | (b) Method of allocation concealment: | Not reported | Not reported | sealed, opaque envelopes in the pharmacy at each site | Central randomization | Not reported | randomised boxes labelled with the centre code and case number |
| (2) **A priori sample size calculation**: | | yes | yes | yes | yes | yes | yes |
| (3) **Blinding:** | (a) Blinding used during the study: | double-blind | double-blind | double-blind for atosiban arms | open-label, no blinding used | double-blind | single-blind |
|  | (b) If used, how was blinded: | double-placebo | double-placebo | double-placebo | NA | double-placebo | double-placebo |
| (4) **Description of withdrawals and dropouts provided:** | | provided | provided | provided | provided | provided | provided |
